# Supplementary material for: Trapping Brown Marmorated Stink Bugs: “The Nazgȗl” Lure and Kill Nets
Source: Insects. 2019 Nov 30;10(12):433. doi: 10.3390/insects10120433 (PMC6955730; doi:10.3390/insects10120433)
Supplement: Supplementary file 1 [file insects-10-00433-s001.pdf]

insects

MDPI

Supplementary Materials

Table S1. Analysis of variance of log-transformed counts of *Halyomorpha halys* using three sampling systems in a hedge row at Fondazione Edmund Mach, Rovereto Farm (Experiment 2).

Effects	DF	Adj SS	Adj MS	F-Value	*p*-Value
Trap	1	22.71	22.71	56.7	<0.001
Method (beating/trap)	1	57.86	57.86	144.6	<0.001
Run	1	6.62	6.62	16.5	<0.001
Traps*Method	1	0.44	1.1	1.1	0.295
Traps*Run	1	5.29	5.29	13.2	<0.001
Method*Run	1	2.79	2.79	7.0	0.009
Traps*Method*Run	1	0.24	0.24	0.6	0.441
Error	232	92.85	0.40		

Table S2. Main effects table for the ANOVA on log-transformed adjusted adult counts of *Halyomorpha halys* in Experiment 3.

Source	DF	Adj SS	Adj MS	F-Value	*p*-Value
Site	2	1.874	0.9368	4.66	0.011
Treatment	1	1.820	1.8205	9.06	0.003
Date	2	2.955	1.4777	7.35	0.001
Error	106	21.305	0.2010		
Lack-of-Fit	12	5.711	0.4759	2.87	0.002
Pure Error	94	15.594	0.1659		
Total	111	27.083			

Table S3. Main effects table for the ANOVA on trap type with log-transformed adjusted adult counts of *Halyomorpha halys* in Experiment 4.

Source	DF	Adj SS	Adj MS	F-Value	*p*-Value
Trap	1	0.6075	0.60755	10.37	0.005
Error	16	0.9377	0.05861		
Total	17	1.5453			
